# Dimethylarginine Dimethylaminohydrolase1 Is an Organ-Specific Mediator of End Organ Damage in a Murine Model of Hypertension

**DOI:** 10.1371/journal.pone.0048150

**Published:** 2012-10-26

**Authors:** Karsten Sydow, Christine Schmitz, Eike-Christin von Leitner, Robin von Leitner, Anna Klinke, Dorothee Atzler, Christian Krebs, Hartwig Wieboldt, Heimo Ehmke, Edzard Schwedhelm, Thomas Meinertz, Stefan Blankenberg, Rainer H. Böger, Tim Magnus, Stephan Baldus, Ulrich Wenzel

**Affiliations:** 1 Department of Interventional Cardiology, Hamburg University Heart Center, University Hospital Hamburg-Eppendorf, Hamburg, Germany; 2 Department of Nephrology, University Hospital Hamburg-Eppendorf, Hamburg, Germany; 3 Institute of Clinical Pharmacology and Toxicology, University Hospital Hamburg-Eppendorf, Hamburg, Germany; 4 Institute of Cellular and Integrative Physiology, University Hospital Hamburg-Eppendorf, Hamburg, Germany; 5 Department of Neurology, University Hospital Hamburg-Eppendorf, Hamburg, Germany; Julius-Maximilians-Universität Würzburg, Germany

## Abstract

**Background:**

The endogenous nitric oxide synthase inhibitor asymmetric dimethylarginine (ADMA) is an independent predictor of cardiovascular and overall mortality. Moreover, elevated ADMA plasma concentrations are associated with the extent of hypertension. However, data from small-sized clinical trials and experimental approaches using murine transgenic models have revealed conflicting results regarding the impact of ADMA and its metabolizing enzyme dimethylarginine dimethylaminohydrolase (DDAH) in the pathogenesis of hypertension.

**Methodology/Principal Findings:**

Therefore, we investigated the role of ADMA and DDAH1 in hypertension-induced end organ damage using the uninephrectomized, deoxycorticosterone actetate salt, and angiotensin II-induced hypertension model in human DDAH1 (hDDAH1) overexpressing and wild-type (WT) mice. ADMA plasma concentrations differed significantly between hDDAH1 and WT mice at baseline, but did not significantly change during the induction of hypertension. hDDAH1 overexpression did not protect against hypertension-induced cardiac fibrosis and hypertrophy. In addition, the hypertension-induced impairment of the endothelium-dependent vasorelaxation of aortic segments *ex vivo* was not significantly attenuated by hDDAH1 overexpression. However, hDDAH1 mice displayed an attenuated hypertensive inflammatory response in renal tissue, resulting in less hypertensive renal injury.

**Conclusion/Significance:**

Our data reveal that hDDAH1 organ-specifically modulates the inflammatory response in this murine model of hypertension. The lack of protection in cardiac and aortic tissues may be due to DDAH1 tissue selectivity and/or the extent of hypertension by the used combined model. However, our study underlines the potency of hDDAH1 overexpression in modulating inflammatory processes as a crucial step in the pathogenesis of hypertension, which needs further experimental and clinical investigation.

## Introduction

The prevalence and morbidity of hypertension is increasing constantly. Despite great achievements in blood pressure therapy, the underlying pathophysiology is still not fully understood. Intriguingly, hypertensive end organ damage is progressing irrespective of the decline in blood pressure. Endothelium-derived nitric oxide (NO) plays a crucial role in the regulation of vascular tone, platelet activity, leukocyte adhesion, and the development of arteriosclerosis [1]. The endogenous NO synthase inhibitor asymmetric dimethylarginine (ADMA) has emerged as an independent predictor of cardiovascular and overall mortality [2]–[4]. Elevated ADMA plasma concentrations are associated with the incidence of hypertension. There is considerable clinical and experimental evidence that NO deficiency develops as a result of chronic kidney disease and is linked to progression of renal dysfunction [5], [6]. Elevations in plasma ADMA have been observed in various pathologies including renal disease, with the highest plasma ADMA concentrations being associated with the most rapid chronic kidney disease progression [7]–[10]. This may be explained by the fact that in animal studies, chronic NO synthase inhibition causes systemic and glomerular hypertension with consecutive glomerulosclerosis, tubulointerstitial injury, and proteinuria [11].

The major degrading pathway for ADMA is its metabolism by the enzyme dimethylarginine dimethylaminohydrolase (DDAH). The DDAH enzyme exists in two different isoforms (DDAH1 and DDAH2) with distinct tissue selectivity [12], [13]. Recently, a mouse overexpressing the human DDAH1 gene has been generated [14]. In these mice, ADMA plasma concentrations are 30–50% lower compared to wild-type (WT) littermates. In addition, hDDAH1 overexpression resulted in improved endothelial regeneration after endothelial denudation, reduced myocardial reperfusion injury, and increased insulin sensitivity [15]–[17]. However, no beneficial effects have been found in a model of ischemic stroke [18]. Interestingly, we were able to detect an interaction between ADMA/DDAH and the leukocyte-derived hemoprotein myeloperoxidase suggesting an important role of ADMA and/or DDAH in regulating inflammatory cascades in cardiovascular diseases [19].

So far, data from small-sized clinical trials and experimental approaches using murine DDAH transgenic and knock-out models have revealed controversial results regarding the impact of ADMA and its metabolizing enzyme DDAH in the development of hypertension. Transgenic mice are a valuable tool for studying the underlying mechanisms leading to hypertensive end organ injury. C57Bl/6 mice serve as a background strain for most of the transgenic mice. However, these mice appear to be resistant to hypertension-induced renal and cardiovascular diseases [20]. In particular, these mice are resistant against models of renal injury, i.e. angiotensin II (Ang II) infusion, protein overflow, or renal ablation [21]–[23]. Hence, we developed a new model of hypertension by combining deoxycorticosterone acetate (DOCA) salt and Ang II infusion [20]. This model does not resemble the pathophysiology of essential hypertension in humans but rather shows substantial hypertensive renal and cardiac injury with concomitant albuminuria, glomerular sclerosis as well as cardiac hypertrophy and fibrosis [20]. Therefore, this model is suitable for studying the effect of overexpression or knockout of genes of interest in hypertensive end organ damage [24].

**Figure 1:**
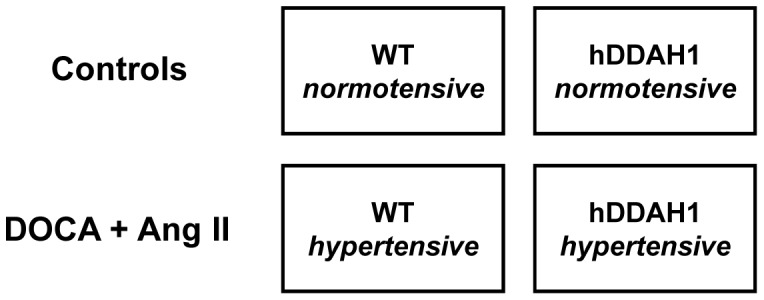
Mouse groups and experimental set-up. Ang II = angiotensin II, DOCA = deoxycorticosterone acetate, hDDAH1 = human dimethylarginine dimethylaminohydrolase1, WT = wild-type.

The aim of the present study was to investigate the role of ADMA and its degrading enzyme DDAH1 in the development of hypertension-induced end organ damage (i.e. aortic, cardiac, and renal tissues) using the recently developed DOCA and Ang II-induced murine hypertension model.

**Table 1:** Gene expression assays.

Gene	Assay
ANP	Mm 01255748_g1
BNP	Mm 00435303_g1
Collagen III	Mm 01254467_m1
Fibronectin	Mm 012567644_m1
MCP-1	Mm 00441242_m1
PAI-1	Mm 00435858_m1
VCAM-1	Mm 01320970_m1
GAPDH	Mm03302249_g1

ANP = atrial natriuretic peptide, BNP = brain natriuretic peptide, MCP-1 = monocyte chemotactic protein-1, PAI-1 = plasminogen activator inhibitor-1, VCAM-1 = vascular cell adhesion molecule-1, GAPDH = Glyceraldehyde 3-phosphate dehydrogenase.

**Table 2:** Mortality and body weight.

	Mortality	Body weight day 35 [g]
WT_normotensive_	0/12	27.7±0.9
hDDAH1_normotensive_	0/10	29.1±1.0
WT_hypertensive_	9/20	24.1±0.9*
hDDAH1_hypertensive_	7/16	22.2±1.0*

WT = wild-type, hDDAH1 = human dimethylarginine dimethylaminohydrolase 1.

* p<0.01; *Body weight*: normotensive vs. hypertensive hDDAH1 and WT, respectively.

## Methods

### Animals

Human DDAH1 transgenic mice were generated in the laboratory of John P. Cooke at Stanford University, CA, United States [14]. Male hDDAH1 transgenic mice were mated with C57Bl/6J female mice (Charles River Laboratories Germany, Sulzfeld, Germany). Offsprings were screened for transgene expression by PCR analysis using tail DNA as described earlier [14]. All experiments were conducted in male, 8–10 weeks-old, heterozygous hDDAH1 transgenic mice, and age-, sex-, and weight-matched WT littermates. All experiments were conducted according to relevant national and international guidelines (German Animal Welfare Act) and were approved by the local Animal Care and Use Committee (Behörde für Soziales, Familie, Gesundheit und Verbraucherschutz – Lebensmittelsicherheit und Veterinärwesen – G10/011).

**Table 3:** L-arginine, monomethyl-, and dimethylarginines plasma concentrations.

	WT_normotensive_	hDDAH1_normotensive_	WT_hypertensive_	hDDAH1_hypertensive_
ADMA [µmol/L]	0.82±0.04	0.51±0.02*	0.85±0.04	0.57±0.05*
SDMA [µmol/L]	0.18±0.01	0.17±0.01	0.18±0.01	0.22±0.02#
L-NMMA [µmol/L]	0.35±0.02	0.24±0.02*	0.43±0.01*	0.31±0.01*;*
L-arginine [µmol/L]	120.5±16.1	98.7±12.7	126.5±11.2	97.6±20.6

ADMA = asymmetric dimethylarginine, SDMA = symmetric dimethylarginine, L-NMMA = *N*-monomethyl L-arginine, WT = wild-type, hDDAH1 = human dimethylarginine dimethylaminohydrolase 1.

WT normotensive: N = 12, hDDAH1 normotensive: N = 10, WT hypertensive: N = 11, hDDAH1 hypertensive: N = 8.

* p<0.01; ADMA: normotensive WT vs. hDDAH1, hypertensive WT vs. hDDAH1.

# p<0.05; SDMA: hypertensive WT vs. hDDAH1.

* p<0.01; L-NMMA: normotensive WT vs. hDDAH1; hypertensive WT vs. hDDAH1, normotensive WT vs. hypertensive WT; normotensive hDDAH1 vs. hypertensive hDDAH1.

**Figure 2:**
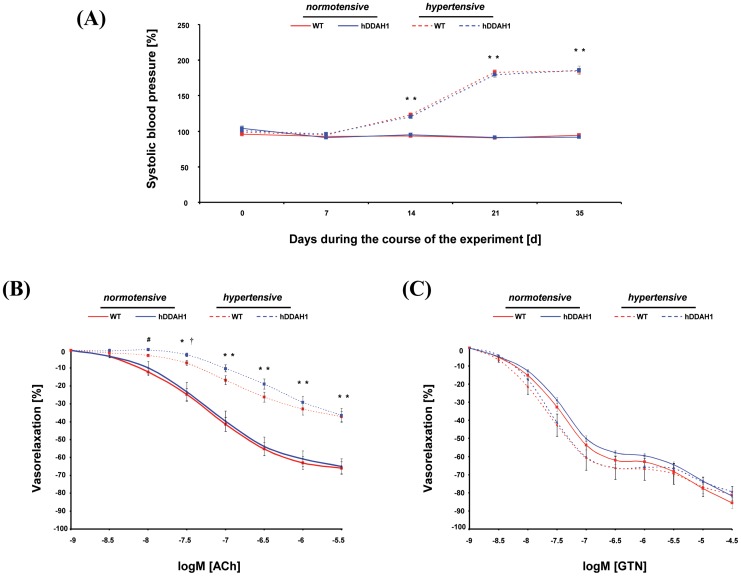
hDDAH1 overexpression does not significantly attenuate the increase of systolic blood pressure and the hypertension-induced impairment of endothelium-dependent vasorelaxation of aortic segments *ex vivo* by the combined hypertensive treatment. (A) Systolic blood pressure during the course of the experiment. Endothelium-dependent (B) and –independent (C) relaxation of aortic segments determined by organ chamber experiments. ACh = acetylcholine, hDDAH1 = human dimethylarginine dimethylaminohydrolase1, GTN = nitroglycerin, WT = wild-type. *ACh and GTN, respectively:* WT normotensive: N = 11, hDDAH1 normotensive: N = 9, WT hypertensive: N = 5, hDDAH1 hypertensive: N = 9. ^*^p<0.01: hDDAH1 hyper- vs. normotensive and/or WT hyper- vs. normotensive. ^#^p<0.05: hDDAH1 hyper- vs. normotensive. ^†^p<0.05: WT hyper- vs. normotensive.

### DOCA + Ang II-induced Hypertension

Hypertension was induced using our recently developed model of DOCA + Ang II-induced hypertension [20]. Briefly, at day 0 mice were uninephrectomized under isoflurane anesthesia and allocated to four different experimental groups (*Figure 1*). Two weeks later, the two hypertensive groups (hDDAH1 and WT hypertensive) received a subcutaneous implantation of a 50 mg DOCA salt pellet (Innovative Research, Sarasota, FL, United States). The DOCA group was given drinking water containing 1% NaCl. At day 21, the hypertensive groups received a subcutaneous implantation of an osmotic minipump (Alzet 1002, Sulzfeld, Germany) delivering 1.5 ng Ang II/min/g body weight (Sigma, Deisenhofen, Germany) for two additional weeks. The animals were sacrificed at day 35.

### Determination of Systolic Blood Pressure

Mice were trained daily for 3 days to have systolic blood pressure (SBP) determined with a computerized tail cuff system (Process Control Blood Pressure 2900-series; TSE Systems, Bad Homburg, Germany). Blood pressure was measured in conscious mice by tail-cuff plethysmography, requiring three measurements per animal for each recording as described recently [20].

**Figure 3:**
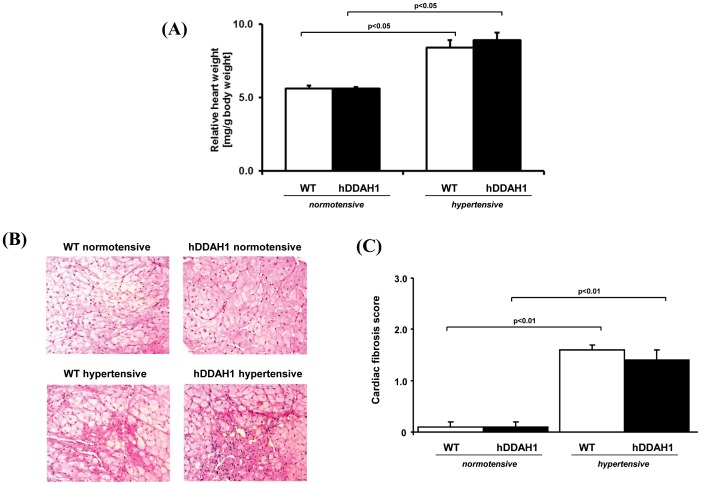
Relative heart weight and histological determination of cardiac changes. (A) Post mortem relative heart weight. (B) Histological examinations revealed extensive fibrosis and scaring in hypertensive hDDAH1 and WT mice with no significant differences between the two hypertensive groups (PAS; original magnification×200). (C) Semiquantitative analysis of the fibrotic cardiac lesions. hDDAH1 = human dimethylarginine dimethylaminohydrolase1, WT = wild-type. WT normotensive: N = 11, hDDAH1 normotensive: N = 10, WT hypertensive: N = 10, hDDAH1 hypertensive: N = 8.

### Blood and Tissue Sampling

Blood was drawn from the abdominal aorta under isoflurane anesthesia at day 35. After systemic application of heparin, the kidneys, heart, and thoracic aorta were carefully removed and placed into phosphate-buffered saline solution. The ventricular heart weight was determined, kidney and heart tissues were fixed in 4% buffered formaldehyde, and aortic segments were prepared for *ex vivo* organ chamber experiments by cleaning of excessive adventitial tissue. For RNA analysis, renal cortex and heart tissue were stored at -20°C until final analyses.

### Determination of Plasma Dimethylarginines

The blood was transferred to 1.5 ml Eppendorf tubes prepared with 10 µl EDTA, immediately centrifuged at 4°C (10 min at 4,000 rpm), and the supernatant was stored at −80°C. ADMA and SDMA plasma concentrations were measured by liquid chromatography-tandem mass spectrometry using isotope-labeled [^2^H_6_]-ADMA as internal standard for quantification of ADMA and SDMA [25].

### Organ Chamber Experiments

Endothelium-dependent (acetylcholine; ACh) and -independent (nitroglycerin; GTN) vasodilator responses were assessed with endothelium-intact, isolated murine aortic rings mounted for isometric tension recordings in organ chambers as described recently [26], [27]. Briefly, intact vascular rings of the thoracic aorta were cut into 4 mm segments, mounted and kept in carbogen-gased, phosphate buffer-filled organ chambers. After preconstriction with prostaglandin F_2α_ (15 µl 10^−2^ M in 25 ml buffer) to achieve 50–80% of the maximal KCl-induced tone, increasing concentrations of GTN (10^−9^ M to 10^−4.5^ M) or ACh (10^−9^ M to 10^−5.5^ M) were added to determine endothelial function.

**Figure 4:**
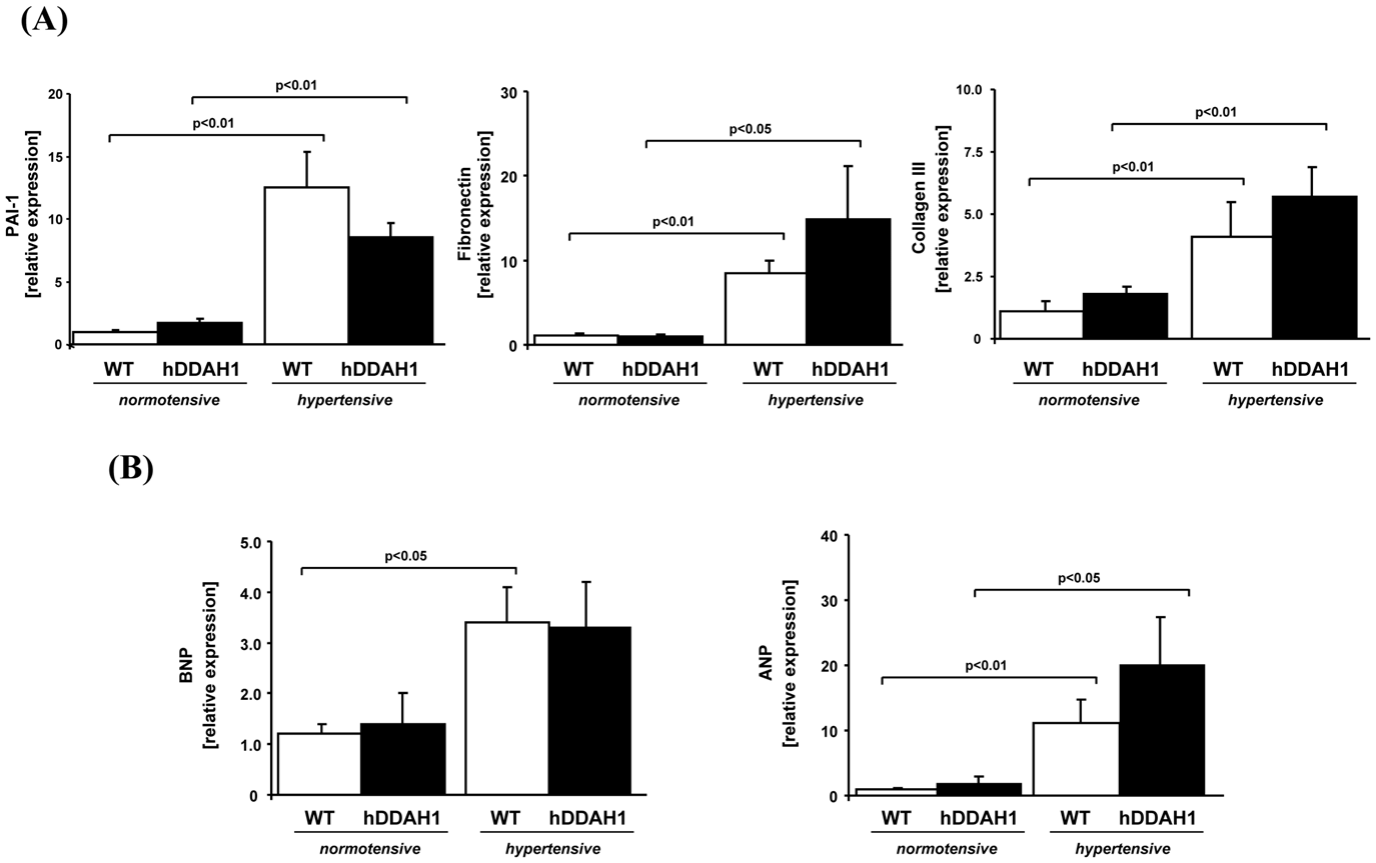
In cardiac tissue, hDDAH1 overexpression does not protect against hypertension-induced hypertrophy and fibrosis. (A) Cardiac profibrotic and hypertrophic markers: determination of murine plasminogen activator inhibitor-1 (PAI-1), fibronectin, and collagen III gene expression in heart tissue. hDDAH1 = human dimethylarginine dimethylaminohydrolase1, WT = wild-type. *PAI-1:* WT normotensive: N = 11, hDDAH1 normotensive: N = 9, WT hypertensive: N = 9, hDDAH1 hypertensive: N = 7. *Fibronectin:* WT normotensive: N = 12, hDDAH1 normotensive: N = 9, WT hypertensive: N = 10, hDDAH1 hypertensive: N = 9. *Collagen III:* WT normotensive: N = 12, hDDAH1 normotensive: N = 10, WT hypertensive: N = 6, hDDAH1 hypertensive: N = 7. (B) Determination of murine brain natriuretic peptide (BNP) and atrial natriuretic peptide (ANP) gene expression in heart tissue. *BNP:* WT normotensive: N = 11, hDDAH1 normotensive: N = 8, WT hypertensive: N = 11, hDDAH1 hypertensive: N = 8. *ANP:* WT normotensive: N = 11, hDDAH1 normotensive: N = 9, WT hypertensive: N = 10, hDDAH1 hypertensive: N = 9.

### Histology of Cardiac and Renal Tissues

Cardiac and renal tissues were stained with periodic acid Schiff (PAS) reagent. Ultrathin sections were stained and photographed using a transmission electron microscope (EM 902; Zeiss, Jena, Germany). For cardiac fibrosis, high-power fields of the right and left ventricles were judged to have no (score = 0), light (score = 1), moderate (score = 2), or severe fibrosis (score = 3). In addition, glomerular injury was determined by qualitative scoring. The score indicated no injury (score = 0), mild injury in less than a quarter of the glomerular tuft (score = 1), damage to more than a quarter of the glomerular tuft (score = 2), and damage to the entire glomerulus (score = 3). Histology scoring in mice was performed in a blinded fashion as established in our laboratory recently [20], [24].

**Figure 5:**
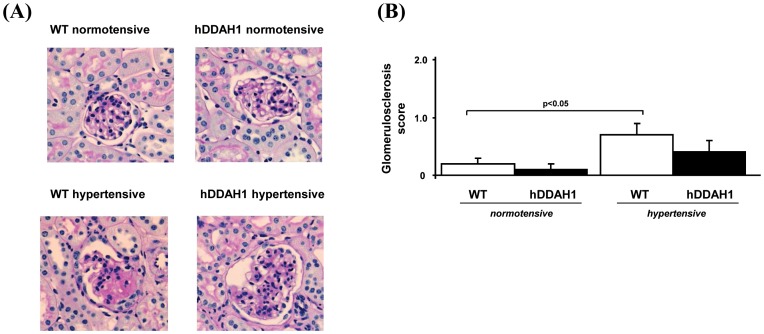
Histological determination of renal changes. (A) Histological examinations with regard to renal injury revealed no significant differences between the two normotensive groups (PAS; original magnification×400). However, hypertensive WT mice showed hypertensive glomerular injury, whereas hDDAH1 mice showed a modest, yet significant, protection against the development of glomerulosclerosis. (B) Semiquantitative analysis of the glomerular injury. hDDAH1 = human dimethylarginine dimethylaminohydrolase1, WT = wild-type. WT normotensive: N = 12, hDDAH1 normotensive: N = 10, WT hypertensive: N = 11, hDDAH1 hypertensive: N = 9.

### Gene Expression Analyses

As markers of cardiac fibrosis, murine plasminogen activator inhibitor-1 (PAI-1), fibronectin, and collagen III were determined in heart tissue. In addition, brain natriuretic peptide (BNP) and atrial natriuretic peptide (ANP) gene expression was assessed in cardiac tissue. In kidney tissue, gene expression of monocyte chemotactic protein-1 (MCP-1) and vascular cell adhesion molecule-1 (VCAM-1) as markers of inflammation beside the profibrotic marker PAI-1 were determined. Total RNA from both ventricles and the kidney cortex was prepared as described previously [28]. Gene expression analyses were determined using standard reverse transcriptase and quantitative TaqMan™ PCR techniques. For RNA preparation (RNAeasy Kit, Qiagen, Hilden, Germany) and cDNA synthesis (cDNA archive kit, Applied Biosystems, Darmstadt, Germany) commercially available kits were used. Prefabricated TaqMan probes (Gene expression assays, Applied Biosystems, Darmstadt, Germany; *Table 1*) were used for final qRT-PCR analysis.

### Determination of Albuminuria

Urine was obtained at the end of the experiment by transferring the mice into metabolic cages for 6 h with access to drinking water *ad libitum*. Albuminuria was measured by sodiumdodecylsulfate gel (10%; Lonza, Cologne, Germany) electrophoresis following Coomassie staining. Blood urea nitrogen and cholesterol were determined using an autoanalyzer (Hitachi 717; Roche, Mannheim, Germany).

### Statistical Analysis

The data are expressed as mean ± standard error of the mean. To test for differences between groups, Student’s unpaired t-test or one-way ANOVA followed by Bonferroni post hoc test was used, as appropriate. A p-value <0.05 was considered as statistically significant.

**Figure 6:**
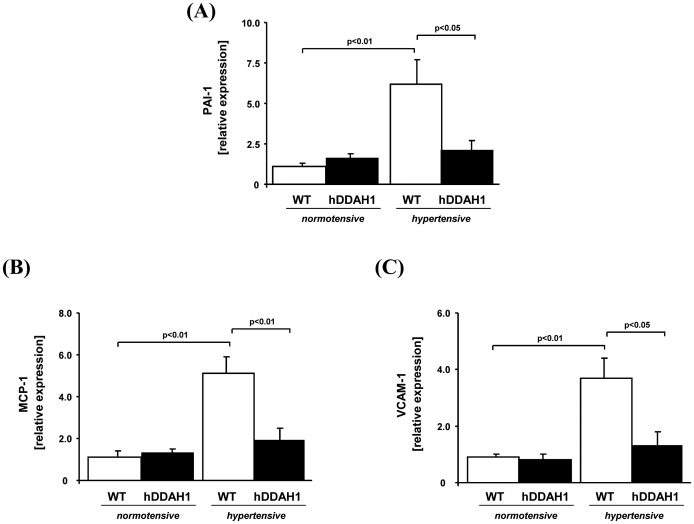
hDDAH1 overexpression is able to attenuate the hypertensive injury in the kidney. (A) Renal profibrotic marker: determination of murine plasminogen activator inhibitor-1 (PAI-1) gene expression in kidney tissue. Renal proinflammatory markers: determination of monocyte chemoattractant protein-1 (MCP-1; B) and vascular cell adhesion molecule-1 (VCAM-1; C) gene expression in kidney tissue. hDDAH1 = human dimethylarginine dimethylaminohydrolase1, WT = wild-type. *PAI-1:* WT normotensive: N = 11, hDDAH1 normotensive: N = 9, WT hypertensive: N = 10, hDDAH1 hypertensive: N = 7. *MCP-1:* WT normotensive: N = 10, hDDAH1 normotensive: N = 9, WT hypertensive: N = 10, hDDAH1 hypertensive: N = 9. *VCAM-1:* WT normotensive: N = 7, hDDAH1 normotensive: N = 7, WT hypertensive: N = 7, hDDAH1 hypertensive: N = 6.

## Results

### Body Weight

The body weight did not significantly differ between the four groups within the first 21 days. By the end of the experiment, a decreased body weight was seen in the two hypertensive groups, without a significant difference between the two hypertensive groups (*Table 2*).

### Dimethylarginines Concentrations

By the end of the study, normo- and hypertensive hDDAH1 mice revealed significantly lower ADMA plasma concentrations as compared to their corresponding WT controls (*Table 3*). Interestingly, DOCA + Ang II did not result in a significant increase of ADMA plasma concentrations, neither in WT nor in hDDAH1 mice. SDMA plasma concentrations did not significantly differ between the two normotensive groups. In contrast to the WT mice, SDMA concentrations significantly increased in hypertensive hDDAH1 mice compared to the normotensive hDDAH1 mice. The L-arginine concentrations in hDDAH1 mice tended to be lower compared to their normotensive and hypertensive WT controls. Interestingly, the induction of hypertension did not result in a significant change of L-arginine concentrations. Normo- and hypertensive hDDAH1 mice revealed significantly lower L-NMMA plasma concentrations as compared to their corresponding WT controls. Interestingly, L-NMMA concentrations were significantly higher in hypertensive hDDAH1 mice compared to hypertensive WT mice at day 35 (hDDAH1 vs. WT hypertensive, 0.31±0.01 vs. 0.24±0.02 µmol/l; p<0.01).

### Systolic Blood Pressure

There was no significant difference in SBP between normotensive hDDAH1 and WT mice (*Figure 2A*). After implantation of the DOCA pellets at day 14, SBP significantly increased in the two hypertensive compared to the normotensive groups (day 19: hDDAH1 hyper- vs. normotensive, 120.7±2.6 vs. 95.1±2.4 mmHg; WT hyper- vs. normotensive, 122.9±1.7 vs. 93.3±1.6 mmHg; each p<0.01). Ang II further increased SBP in the two hypertensive mouse groups at day 26 and 33, without significant differences between WT and hDDAH1 mice.

### Aorta

#### Vasodilator responses in isolated aortic segments

In normotensive mice, the sensitivity of isolated aortic segments to endothelium-dependent and -independent vasodilation showed no significant difference between the groups with regard to the maximum vasodilation and at any other used concentration (*Figure 2B and 2C*). In contrast, hypertensive mice showed a significantly impaired endothelium-dependent vasodilation (normotensive vs. hypertensive hDDAH1; 64.8±4.2 vs. 36.4±3.9% and normotensive vs. hypertensive WT; 65.8±3.6 vs. 37.0±2.8% maximum response; *Figure 2B*; each p<0.01), without any significant changes between the two hypertensive groups. Compared to the normotensive groups the impaired endothelium-dependent response reached statistical significance starting with −8.0 log M [ACh] in hDDAH1 mice and −7.5 log M [ACh] in WT mice. Hypertensive hDDAH1 and WT mice showed no significant impairment of the endothelium-independent vasodilation compared to the corresponding normotensive groups (*Figure 2C*).

**Figure 7:**
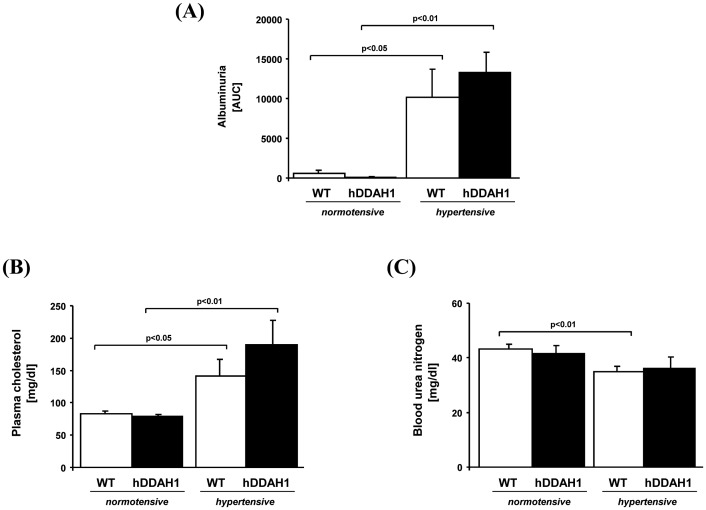
Determination of albuminuria (A) and cholesterol (B) and urea nitrogen (C) plasma concentrations. hDDAH1 = human dimethylarginine dimethylaminohydrolase1, WT = wild-type. *Albuminuria:* WT normotensive: N = 4, hDDAH1 normotensive: N = 4, WT hypertensive: N = 4, hDDAH1 hypertensive: N = 4. *Cholesterol:* WT normotensive: N = 11, hDDAH1 normotensive: N = 9, WT hypertensive: N = 11, hDDAH1 hypertensive: N = 8. *Urea nitrogen:* WT normotensive: N = 10, hDDAH1 normotensive: N = 10, WT hypertensive: N = 11, hDDAH1 hypertensive: N = 8.

### Heart

#### Post mortem relative heart weight and cardiac histology

The relative heart weight was significantly increased in hypertensive mice, with no significant difference between hDDAH1 and WT mice (*Figure 3A*). DOCA salt + Ang II induced cardiac fibrosis in hypertensive mice (*Figure 3B and C* *)*. In both hypertensive groups, histological examinations demonstrated areas of fibrosis and concomitant loss of cardiomyocytes. However, scoring of the fibrosis revealed no significant difference between hypertensive hDDAH1 and WT mice.

#### Gene expression analyses of profibrotic and hypertrophic cardiac markers

Cardiac fibrosis was also assessed by gene expression analysis of PAI-1 (*Figure 4A*; hDDAH1 hyper- vs. normotensive, 4.8 fold increase; WT hyper- vs. normotensive, 12.5 fold increase; each p<0.01), fibronectin (hDDAH1 hyper- vs. normotensive, 14.9 fold increase; p<0.05; WT hyper- vs. normotensive, 7.7 fold increase; p<0.01), and collagen III (hDDAH1 hyper- vs. normotensive, 4.1 fold increase; WT hyper- vs. normotensive, 3.7 fold increase; each p<0.01). Whereas a significant upregulation of each of these gene markers was found in hypertensive mice, no significant difference was seen between hDDAH1 and WT mice. Gene expression analysis of hypertrophic cardiac markers revealed a significant, hypertension-induced increase of BNP in WT mice (*Figure 4B*; WT hyper- vs. normotensive, 2.8 fold increase; p<0.05), whereas the increase of BNP expression in hDDAH1 mice was not significant (hDDAH1 hyper- vs. normotensive, 2.4 fold increase; p = n.s.). In line with the hypertrophic genes, the expression of the fetal gene ANP was upregulated significantly in hypertensive mice (*Figure 4B*; hDDAH1 hyper- vs. normotensive, 11.1 fold increase; p<0.05; WT hyper- vs. normotensive, 11.2 fold increase; p<0.01), but again no significant difference in response to hDDAH1 overexpression was found.

### Kidney

#### Renal histology

DOCA + Ang II induced hypertensive focal and segmental injury with capillary obsolence, plasma insudation, and matrix expansion. Representative micrographs are shown in *Figure 5A*. Semiquantitative analysis of glomerular injury by scoring revealed a significant DOCA + Ang II-induced glomerular injury in hypertensive WT mice as compared to normotensive WT mice (*Figure 5B*). However, in hypertensive hDDAH1 mice the induction of glomerular injury was blunted and not significantly increased compared to normotensive hDDAH1 mice.

#### Gene expression analyses of profibrotic and proinflammatory renal markers

The histology data were corroborated by the analysis of gene expression of profibrotic and inflammatory markers. DOCA + Ang II significantly induced PAI-1 (5.6 fold increase), MCP-1 (4.6 fold increase), and VCAM-1 (4.1 fold increase) in WT mice (*Figure 6A–C*; WT hyper- vs. normotensive, each p<0.01). Strikingly, this induction was significantly blunted in hDDAH1 mice (*Figure 6B*; PAI-1∶1.3 fold increase, MCP-1∶1.5 fold increase, and VCAM-1∶1.6 fold increase; hDDAH1 hyper- vs. normotensive, each p = n.s.).

#### Albuminuria

Moreover, DOCA + Ang II induced severe albuminuria (*Figure 7A*), which was within the nephrotic range as demonstrated by elevated cholesterol plasma concentrations (*Figure 7B*; hDDAH1 hyper- vs. normotensive, 190.0±37.4 vs. 78.4±3.1 mg/dl; p<0.01; WT hyper- vs. normotensive, 141.4±26.0 vs. 83.4±3.8 mg/dl; p<0.05). However, there was no significant difference between the hypertensive groups in terms of albuminuria and plasma cholesterol concentrations. Blood urea nitrogen was measured as a marker of renal function and was slightly, yet significantly decreased in hypertensive compared to normotensive WT mice, suggesting hyperfiltration (*Figure 7C*; WT hyper- vs. normotensive, 34.9±2.1 vs. 43.3±1.6 mg/dl; p<0.01). Interestingly, the slight decrease in urea nitrogen did not reach statistical significance in hypertensive hDDAH1 mice.

## Discussion

Interpretation of clinical studies indicating a relationship between increased ADMA concentrations and kidney disease is complicated by differences in study design, study cohorts, and treatment regimen. Evidence for an association between elevated blood pressure and increased ADMA concentrations is based on small-number clinical studies [29], [30]. However, other reports, including large-scale clinical studies, did not confirm this relationship [3], [31]. The generation of hDDAH1 overexpressing mice facilitates to examine the effects of ADMA on cardiovascular and renal end organ damage [14]. The present study provides evidence on the organ-specific, protective, anti-inflammatory impact of hDDAH1 in hypertension-induced end organ damage. The salient findings are: (i) ADMA plasma concentrations differed significantly between hDDAH1 and WT mice at baseline and after the induction of hypertension, but did not significantly increase upon induction of hypertension. (ii) The hypertension-induced impairment of endothelium-dependent vasorelaxation of aortic segments *ex vivo* was not significantly attenuated by hDDAH1 overexpression. (iii) In cardiac tissue, hDDAH1 overexpression did not protect against hypertension-induced hypertrophy and fibrosis. (iv) hDDAH1 overexpression was able to attenuate the hypertensive injury in the kidney.

### Role of ADMA/DDAH in Regulating Circulation

Enzymatic degradation of ADMA by DDAH1 and 2 provides the majority of ADMA removal. DDAH is widely distributed among the tissues and most abundant in the kidney, liver, and vascular endothelium [32]. It has been suggested that renal and hepatic DDAH1 activity controls plasma ADMA, whereas DDAH2 regulates vascular tissue ADMA and hence, vascular tone without affecting plasma ADMA [11], [32]. However, this seems to be a more simplistic perspective, since recent studies in individuals undergoing single nephrectomy for kidney donation support the evidence of additional modulators of ADMA concentrations besides renal and hepatic DDAH1 and 2 activity [33]. Global hDDAH1 overexpression causes enhanced vascular and cardiac DDAH activity without any increase of renal or hepatic DDAH activities [34]. Nevertheless, plasma ADMA and blood pressure decrease, indicating a role of enhanced cardiovascular DDAH activity in plasma ADMA control in these mice. Recent studies in homozygous DDAH1 knockout mice revealed an approximate 20 mmHg increase in blood pressure [35]. More interestingly, despite normal tissue concentrations and distribution of the DDAH2 enzyme, DDAH activity was not detectable in kidney, liver, and lung tissues of these mice.

### Vasculature

ADMA has been shown to be associated with endothelial dysfunction. Infusion of exogenous ADMA increases systemic vascular resistance, elevates mean arterial pressure, reduces cardiac output, and augments pulmonary vascular resistance in men, demonstrating a causal relationship between increased ADMA concentrations and cardiovascular dysfunction [36], [37]. In addition, ADMA administration dose-dependently impairs renal blood flow, sodium reabsorption, and increases vascular stiffness [36]. Selective vascular endothelial DDAH1 knock out (endo-DDAH1^−/−^) mice demonstrate increased ADMA plasma concentrations and blood pressure and exhibit a significantly attenuated acetylcholine-induced NO production and vessel relaxation in isolated aortic rings [38]. However, we have recently shown that isolated aortic segments from hDDAH1 transgenic mice did not show any significant changes in endothelium-dependent or –independent vasodilator response under baseline physiological conditions *ex vivo* [34]. In our present study, the endothelium-dependent vasodilator response was significantly impaired in aortic segments of hypertensive animals and hDDAH1 transgenicity did not exert protective effects.

In addition, the hypertension-induced by DOCA + Ang II did not result in a significant increase of ADMA plasma concentrations, neither in WT nor in hDDAH1 mice, despite profound hemodynamic effects and severe end organ damage. This finding is consistent with the observations by other groups [39], [40]. Sasser et al. demonstrated that chronic infusion of Ang II at 200 ng/kg/min for up to 3 weeks was not sufficient to increase circulating or kidney cortex ADMA concentrations in the rat, despite increased blood pressure, whereas with 6 weeks of high dose Ang II at 400 ng/kg/min an increase in plasma ADMA concentrations but no change in renal cortex ADMA content was observed [40]. The induction of endothelial dysfunction despite a lack of increase in ADMA plasma concentrations, may suggest ADMA-independent adverse effects on the endothelium (i.e. Ang II-induced production of reactive oxygen species). In addition, SDMA may indirectly affect NO synthesis by interacting with the y^+^ transporter that mediates the intracellular uptake of L-arginine and methylarginines and thereby, impairing renal tubular L-arginine absorption [41], [42].

### Heart

The presence of left ventricular hypertrophy (LVH) in hypertension is associated with an increased risk of mortality and morbidity additive to the risk of hypertension. Hypertension has been considered as the main cause of LVH, however, up to 60 percent of the variance of LV mass may be due to other factors independent of blood pressure [43]. In our present study, DOCA + Ang II induced severe cardiac injury with increased hypertrophy, fibrosis, and expression of fetal genes and matrix components. Local upregulation of Ang II protein and activity plays an important role in LVH development. Recent evidence suggests that a healthy coronary microvascular endothelium opposes this effect by serving as a paracrine source of NO, a physiological antagonist of Ang II activity, and that upregulation of this mechanism may account for the protective role of bradykinin with respect to LVH [44]. Due to the association between arterial and cardiac remodeling with altered endothelial microcirculatory, interference of ADMA and/or DDAH with the NO system may be relevant for the pathogenesis of LVH [45]. In our present study, hDDAH1 overexpression did not protect hypertensive animals from increasing LV mass and fibrosis. Considering the results of our study and the work by others, overexpression of DDAH1 does not seem to play a significant role in the context of hypertension-induced cardiac end organ damage. Failure to ameliorate endothelial dysfunction and subsequent hypertensive cardiac disease is in line with our recent observation that hDDAH1 mice are not protected from ischemic stroke [18].

### Kidney

The kidney is a target for hypertensive end organ damage. DOCA + Ang II induce glomerulosclerosis and albuminuria within the nephrotic range. We used a number of quantitative and semiquantitative measures to gauge the extent of renal injury in hDDAH1 and WT mice. The injury was assessed in PAS-stained renal sections by semiquantative scoring of glomerular damage and confirmed by upregulation of PAI-1, MCP-1, and VCAM-1 gene expression, indicating an enhanced renal damage in both hypertensive mice. Intriguingly, hypertensive hDDAH1 mice showed significantly less glomerular injury and expression of fibrotic and inflammatory markers compared to hypertensive WT mice, suggesting that hDDAH1 overexpression protects against hypertensive renal injury. This nephroprotective effect of hDDAH1 overexpression occurred despite any significant changes in plasma ADMA concentrations, which is consistent with the findings by Jacobi et al. [39].

Activation of inflammatory cells, i.e. macrophages and neutrophils plays an important role in the development of glomerulosclerosis and renal fibrosis. MCP-1 is one of the most prominent chemokines that regulates monocyte-macrophage infiltration. In mice lacking the MCP-1 receptor (CCR2^−/−^), Ang II-induced vascular inflammation and remodeling were significantly reduced [46]. The organ-specific, protective effect of hDDAH1 overexpression in our present study, underlines the results by Liao et al. [47]. These investigators revealed that chronic Ang II infusion caused similar increases in SBP and LVH in CCR2^−/−^ and WT mice, whereas nitrotyrosine concentrations as a marker of oxidative stress in renal tissue, macrophage infiltration, albuminuria, and renal damage were significantly decreased in kidneys of CCR2^−/−^ mice compared with age-matched WT mice [47]. Studies using the hDDAH1 mouse model discovered the role of ADMA and/or DDAH in modulating inflammatory processes in a variety of clinical conditions, i.e. transplant vasculopathy, myocardial reperfusion injury, and endothelial regeneration [15], [16], [48]. Recently, we were able to detect a pathogenic vicious cycle between ADMA/DDAH and the leukocyte-derived myeloperoxidase, resulting in neutrophil activation and degranulation, DDAH inactivation, and further ADMA accumulation [19]. This interaction and proinflammatory modulation may contribute to the initiation of cardiovascular and renal diseases. As a result of the attenuated inflammatory response, hypertensive hDDAH1 showed a modest, yet significant, protection against the development of glomerulosclerosis in our present study. We found no significant differences with regard to albuminuria between hypertensive hDDAH1 and WT mice indicating that the protective mechanism of hDDAH1 acts differentially on albuminuria and glomerulosclerosis. The data corroborate the finding by Matsumoto et al. that adenovirus-mediated overexpression of DDAH1 significantly reduced glomerular and interstitial fibrosis in subtotally nephrectomized rats [49].

### Limitations of the Study

Besides a possible lack of hDDAH1 in protecting the heart muscle from hypertension-induced end organ damage, one may consider that the combined hypertensive treatment with Ang II and DOCA infusion in our present study may have led to a strong additive effect, which could not be prevented by hDDAH1 overexpression. Besides Ang II, aldosterone is an important independent mediator of cardiac damage, since spironolactone – an aldosterone antagonist – may prevent the development of cardiac fibrosis [50]. Interestingly, this spironolactone effect was independent of the development of LVH and elevated SBP. The blood pressure results of our present study may be limited, since we used the non-invasive tail-cuff method. Although we may have missed subtle blood pressure differences between genotypes with this technique, the overall effect of Ang II treatment on blood pressure was clearly evident and within the range reported by others. Moreover, no significant differences were found with regard to the relative heart weight between the two hypertensive groups supporting our findings that no major differences with regard to blood pressure occurred.

We did not determine tissue ADMA concentrations. This issue may be of interest and should be focused in further experimental studies, since tissue ADMA concentrations may have been affected by Ang II in contrast to plasma ADMA concentrations. In this particular study, we did not determine creatinine values. However, in addition to determining albuminuria, plasma cholesterol, and urea nitrogen concentrations, we measured SDMA plasma concentrations, which are known to be an excellent marker of renal function [51].

Previously, we have shown that the hDDAH1 transgene is highly expressed among different tissues (i.e. kidney, heart, and aorta) [34]. However, despite the highest hDDAH1 expression in heart tissue we did not observe a protective anti-hypertensive effect in the present study. Therefore, the lack of cardiac protection in hDDAH1 transgenic mice is not driven by a low cardiac expression of the transgene but more likely caused by a different pathophysiology (i.e. additional ADMA independent effects) with regard to hypertensive end organ damage in the kidney and the heart.

### Conclusion

In conclusion, our data reveal an organ-specific potency of hDDAH1 overexpression in protecting hypertensive end organ damage. The lack of protection in cardiac and aortic tissue may be due to DDAH1 tissue selectivity and/or the strong induction of end organ damage by the model used. In the kidney, our data reveal nephroprotective, anti-inflammatory properties of DDAH1 overexpression and therefore, underscore the potential of ADMA being an important modulator in hypertensive kidney disease. We believe that understanding the underlying mechanisms by which DDAH and ADMA affect renal injury may open a new avenue for treatment strategies in hypertension. Substitution of DDAH1 protein or enhancement of its activity may become a novel treatment strategy for renal diseases.
